# Effects of internal migrants' health on economic resilience in China's Yangtze River Delta urban agglomeration: moderating effects of basic public health services

**DOI:** 10.3389/fpubh.2024.1392657

**Published:** 2024-05-07

**Authors:** Shengzhi Zhang, Yanlong Deng

**Affiliations:** ^1^School of Public Administration, Shanghai Open University, Shanghai, China; ^2^School of Social and Public Administration, East China University of Science and Technology, Shanghai, China

**Keywords:** internal migrants, health performance, economic resilience, basic public health services, Yangtze River Delta urban agglomeration

## Abstract

**Introduction:**

Internal migrants constitute a significant generality in the socioeconomic development of developing countries. With the frequent occurrence of major public health emergencies, obstacles to labor supply due to health issues among internal migrants not only affect their livelihood stability but also urban economic resilience. Moreover, the design of basic public health service systems tends to favor local residents over internal migrants, further exacerbating the health and employment risks of internal migrants. As a result, urban economic resilience faces significant challenges.

**Objective:**

The objective of this study was to deconstruct economic resilience into economic resistance and recovery abilities, investigate the net effect and its heterogeneity of internal migrants' health on economic resilience in China's Yangtze River Delta urban agglomeration (CYRD), and the mediating effect from labor participation rate and labor time supply, as well as the moderating effect of basic public health services.

**Methods:**

Based on the China Migrants Dynamic Survey data (CMDS), the study empirically estimated the effects of internal migrants' health on economic resilience in CYRD through microeconometric analysis methods, mediating and moderating effect model.

**Results:**

Our findings indicate that internal migrants' health has a positive effect on economic resilience in CYRD. For each unit increase in migrants' health, it will drive up the average economic resistance ability by 0.0186 and the average recovery ability by 0.0039. Secondly, the net effects of migrants' health on economic resilience show significant structural differences, industry and city heterogeneity. The effect of migrants' health on economic resistance ability is significantly higher than that on economic recovery ability; The effect of migrants' health on economic resilience of the secondary industry is higher than that of the tertiary industry; The cities with high economic resistance and recovery abilities have more prominent positive effect from migrants' health. Thirdly, migrants' health not only has a direct effect on the economic resistance and recovery abilities, but also has a mediating effect on which through labor participation rate and labor time supply.

**Discussion:**

Enhancing the accessibility and quality of basic public health services is beneficial for enhancing the positive effects of internal migrants' health on economic resilience.

## 1 Introduction

Economic resilience is an important focus for urban agglomerations to address challenges from internal and external environmental changes and to move toward high-quality economic development (1). It is also a key support to avoid a hard landing of the national economy and to solidify the foundation for economic recovery under the pressure of a global macroeconomic downturn. As the main carriers of urbanization and national economic, also the strategic pivot of national economic development and regional development strategy, urban agglomerations is conducive to breaking administrative barriers, enhancing industrial agglomeration and connectivity (2), thus accelerating the aggregation of population and factor resources, providing employment opportunities, and promoting regional economic development (3). However, with risks such as natural disasters, economic crises, trade frictions, and major public health incidents continuously accumulating, urban agglomerations has entered a period of frequent internal and external shocks (4, 5). The ability of urban agglomerations to provide a endogenous driving force for regional and national economic development is facing great challenges (6). In this grim reality, economic resilience can help the economic entity withstand external shocks and recover quickly (7). Therefore, it becomes crucial for urban agglomeration economic systems to break through predicaments, get rid of the downward economic trajectory, and achieve steady economic growth.

Internal migrants also provides important support for urbanization advancement and social-economic development, and it helps promote factor agglomeration, industrial transfer, and equalization of public services, driving balanced regional economic development and social integration (8). The laws of world economic development indicate that the economic takeoff of any country inevitably accompanies the process of urbanization, which is particularly prominent in China as a populous and economically influential country (9). According to China's population census data, urbanization rate increased from 36.5% in 2000 to 63.89% in 2020, with the urban population increasing from 460 million to 900 million.^1^ Among them, the contribution of population mobility to the increase in urbanization level is about 45%, higher than the contribution of urban areas expansion, which accounts for 35% of the urbanization rate.^2^ According to the United Nations *World Population Prospects*' forecast, by 2030, China's urbanization rate will reach about 71%, corresponding to 1.03 billion urban residents, an increase of about 130 million from 2020. About 50% of the newly added urban population comes from rural-urban migration, and about 80% of the new urban population will be distributed in 19 major urban agglomerations.^3^ Moreover, this spontaneous migration of population between rural and urban areas driven by market forces has shown certain positive effects in optimizing social resource allocation and regional technological exchanges (10), stimulating consumption (11), and narrowing regional income gaps (12), and has been recognized by domestic and foreign scholars. It can be seen that such large-scale population mobility is not only an important factor affecting China's urbanization, industrialization, and modernization but also a major variable that cannot be ignored in the development of regional relations.

However, as an important part of the economic development of urban agglomerations, the risk resistance ability of the internal migrants is much lower than that of the local population when facing internal and external shocks to cities. Taking public health emergencies as an example, the sudden outbreak of the COVID-19 pandemic in 2021 brought about huge economic losses and employment shocks to countries around the world (13, 14), including China. And the restrictions on labor mobility caused by epidemic prevention measures led to a continuous expansion of the gap between labor supply and demand in cities, resulting in a sharp increase in the unemployment rate among migrant workers (15, 16). China's urban labor market is also facing unprecedented pressure, with difficulties in recruiting workers and employment being reinforced. The temporary and informal employment for migrant workers makes the risks of unemployment and disease particularly severe (17). The hindrance to labor supply caused by health problems among migrant workers not only affects their own livelihood stability but also affects the sustainability and resilience of urban economic development (18). Moreover, the design of an urban public health service system tends to favor local urban residents rather than migrant workers, further exacerbating the health and employment risks of migrant workers, thus posing significant challenges to the resilience of urban economic development. Therefore, systematically exploring the impact of migrant workers' health on the economic resilience of CYRD and the regulatory effects from public health services not only provides new perspectives for studying the economic resilience, but also brings breakthroughs for enhancing economic resilience.

Existing literature closely related to this study mainly includes two branches. The first branch explores the disadvantaged position of migrant workers in terms of health and employment. For a long time, a large number of studies on migrant workers in the United States have pointed out a significant positive correlation between low socioeconomic status and health disadvantages among non-Hispanic whites and blacks (19). A series of studies based on the theory of dual labor markets also indicate that migrant workers, especially those who move across national borders, tend to engage in dangerous and demeaning work (20). Furthermore, research suggests that due to disadvantages such as lack of medical insurance, migrant workers tend to return to their places of origin after falling ill, which may reduce the labor supply in destination cities and the social welfare of migrant workers themselves (21). The second branch discusses the factors influencing economic resilience. Factors such as centrality and diversity of industrial linkages and entrepreneurial vitality are important factors affecting urban economic resilience (22). For example, industrial diversification can provide a favorable environment for technological innovation, effectively promote the enhancement of urban innovation capability and structural adjustment, and thereby enhance urban economic resilience (23). Enhanced innovation capability can also effectively drive the improvement of urban economic resilience (24), mainly through strengthening talent reserves, upgrading industrial structure, and optimizing resource allocation efficiency (25). In addition, existing research have explored the effect of different factors on economic resilience from various aspects such as psychological expectations (26), labor skills (27), economic agglomeration level (28), economic structure (29), transportation infrastructure (30), and spatial structure (31). In summary, existing literature has conducted a series of studies on migrant workers' health and economic resilience separately, but literature attempting to explore the relationship between the two is rare.

This study selects the CYRD, one of the six largest urban agglomerations in the world and the main gathering place of internal migrants in China, as a typical case. According to China's national population census in 2020, the total number of internal migrants in CYRD is 73.573 million, accounting for 19.6% of the country's internal migrants and ~42% of the total local population. It can be said that migrants constitute the backbone of the local labor systems (LLS) in CYRD, serving as the main force driving its socioeconomic development and urban construction. Based on micro-sample data from CMDS, this paper investigates the effect of internal migrants' health on the economic resilience of CYRD, the mechanisms of action, and the policy regulatory effects. The marginal contribution are 2-fold: firstly, it decomposes economic resilience into economic resistance and recovery abilities, explores the factors influencing urban agglomeration economic resistance and recovery abilities from the perspective of migrant workers' health in an innovative way, expands the research scope of factors influencing urban agglomeration or urban economic resilience, and opens up new perspectives for the study of economic resilience. Secondly, it systematically analyzes the logical mechanisms by which migrant workers' health affects the economic resilience from the dimensions of labor participation rate and labor supply, and further explores the regulatory effects of urban public health services on migrant workers' health and the economic resilience, providing new ways for enhancing the economic resilience.

**Figure 1:**
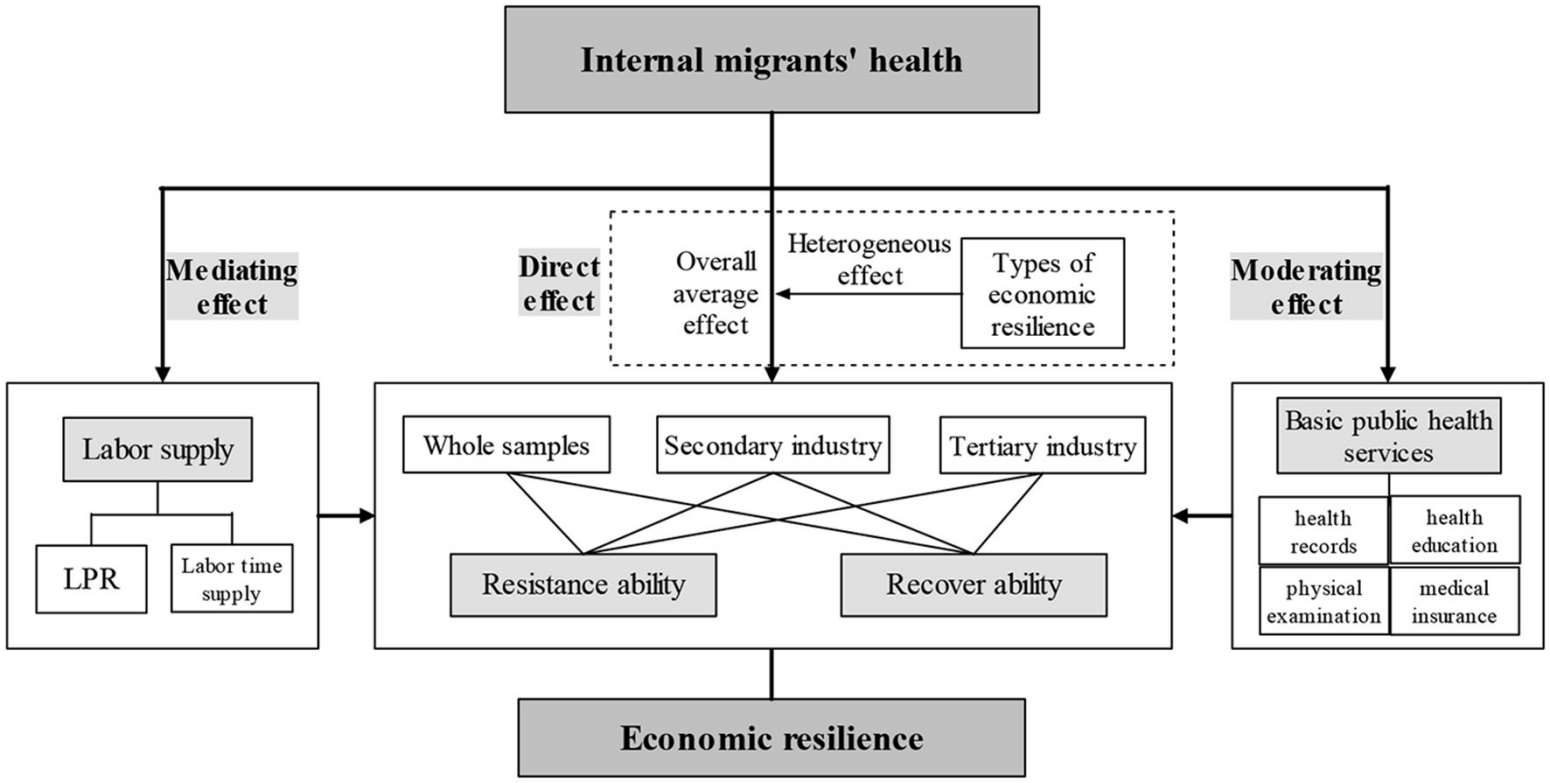
A research framework for this study.

This study is structured as follows: the identification method for economic resilience and the identifying equations of internal migrants' health on economic resilience are presented in Section 2. The regression estimation results and the results of the mediating and moderating effects tests are presented in Section 3. Section 4 draws conclusions and policy implications. The research framework is shown in Figure 1.

## 2 Methodology

### 2.1 Case

**Figure 2:**
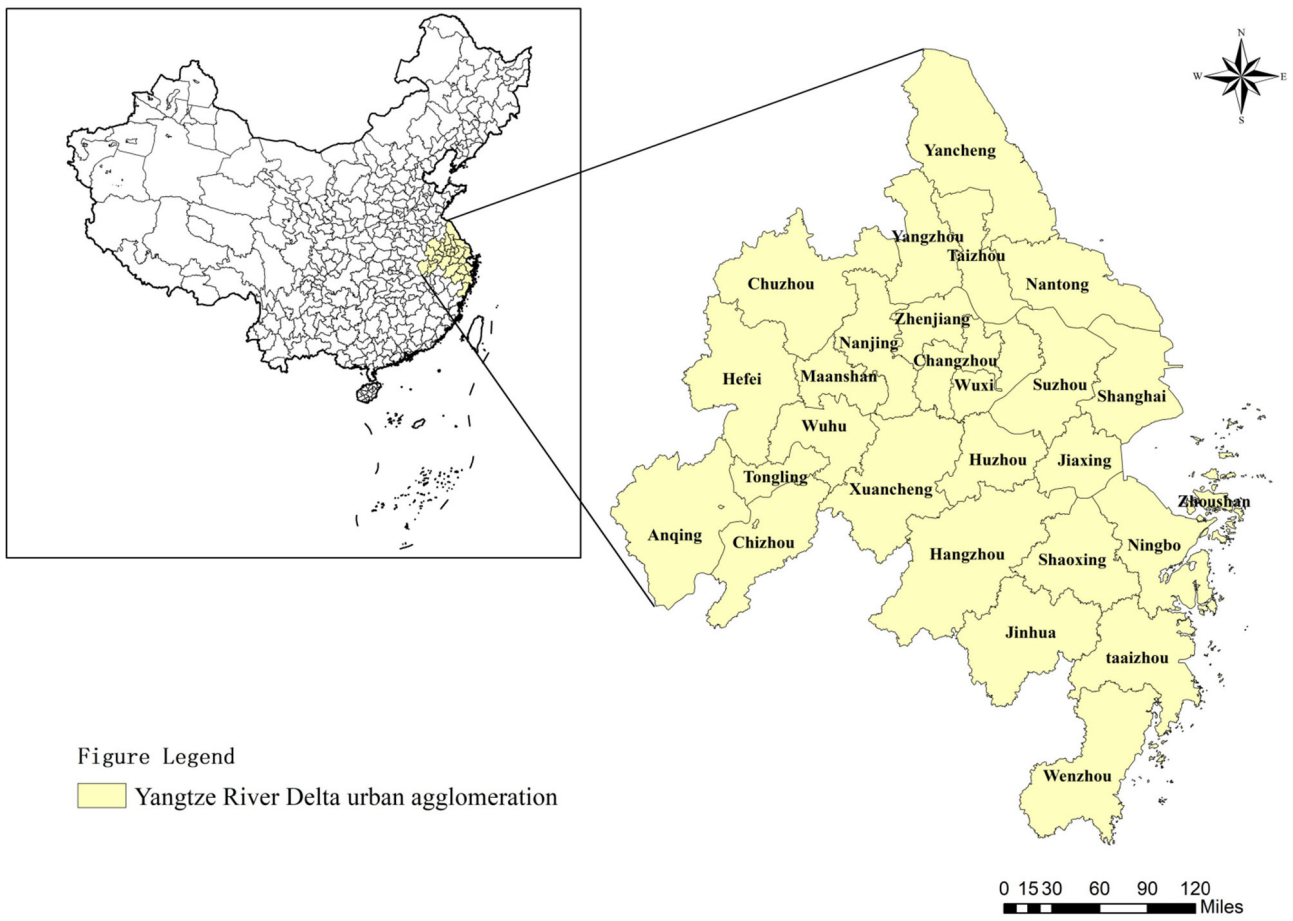
The spatial distribution of the samples in this study.

As one of the fastest-growing regions in both China and the world in terms of economic growth and urbanization, CYRD plays a demonstrative role in the construction of urban agglomerations in China. It holds significant strategic importance in opening-up development and modernization construction, and provides a lot of replicable and promotable experiences for the development of other regions. At present, CYRD includes Shanghai and 26 other cities from Jiangsu, Zhejiang, and Anhui. In Figure 2, CYRD covers an area accounting for about 2.2% of China's territory. However, it hosts ~16.7% of China's population, nearly 25% of its total economic output, and 33.3% of its total import and export volume. Therefore, studying the impact of migrants' health on the economic resilience of CYRD has significant practical implications.

### 2.2 Economic resilience identifying

Economic resilience refers to the ability of an economy to effectively respond to external disturbances, resist risk shocks, and achieve economic autonomy and sustainable development. From existing literature, there appear to be two main methods for identifying economic resilience (32, 33). One approach involves constructing a multidimensional indicator system for economic resilience, assigning weights to each indicator, and then calculating a composite index of urban economic resilience (34). The other approach is to build a sensitivity index based on core variables to identify urban economic resilience (35, 36). Due to the lack of universally recognized indicators and weights for economic resilience in the academic community, as well as the lack of precision and universality in the indicator systems constructed for measuring the economic resilience of different types of regions, this study adopts the approach of Faggian et al. (33) to construct a Sensitivity Index (*SI*) based on the core indicator of employment level for identifying urban economic resilience.


(1) $$\begin{array}{l} SI_{it}=\frac{employment_{i,t}/employment_{i,t-k}}{employment_{n,t}/employment_{n,t-k}} \end{array}$$

Where *employment*_*i, t*_ is total employment in city *i* during period *t*, *employment*_*n, t*−*k*_ is total employment in the country during period *t*−*k*. The *SI* index is similar to the location entropy index and is centered around 1. When the *SI* is > 1, it indicates that the employment growth of city i is faster compared to the national average during the *t*−*k* to *t* period, suggesting relatively strong economic resilience. When the *SI* is < 1, it indicates that the employment growth of city i is slower compared to the national average during the *t*−*k* to *t* period, indicating relatively weak economic resilience.

**Figure 3:**
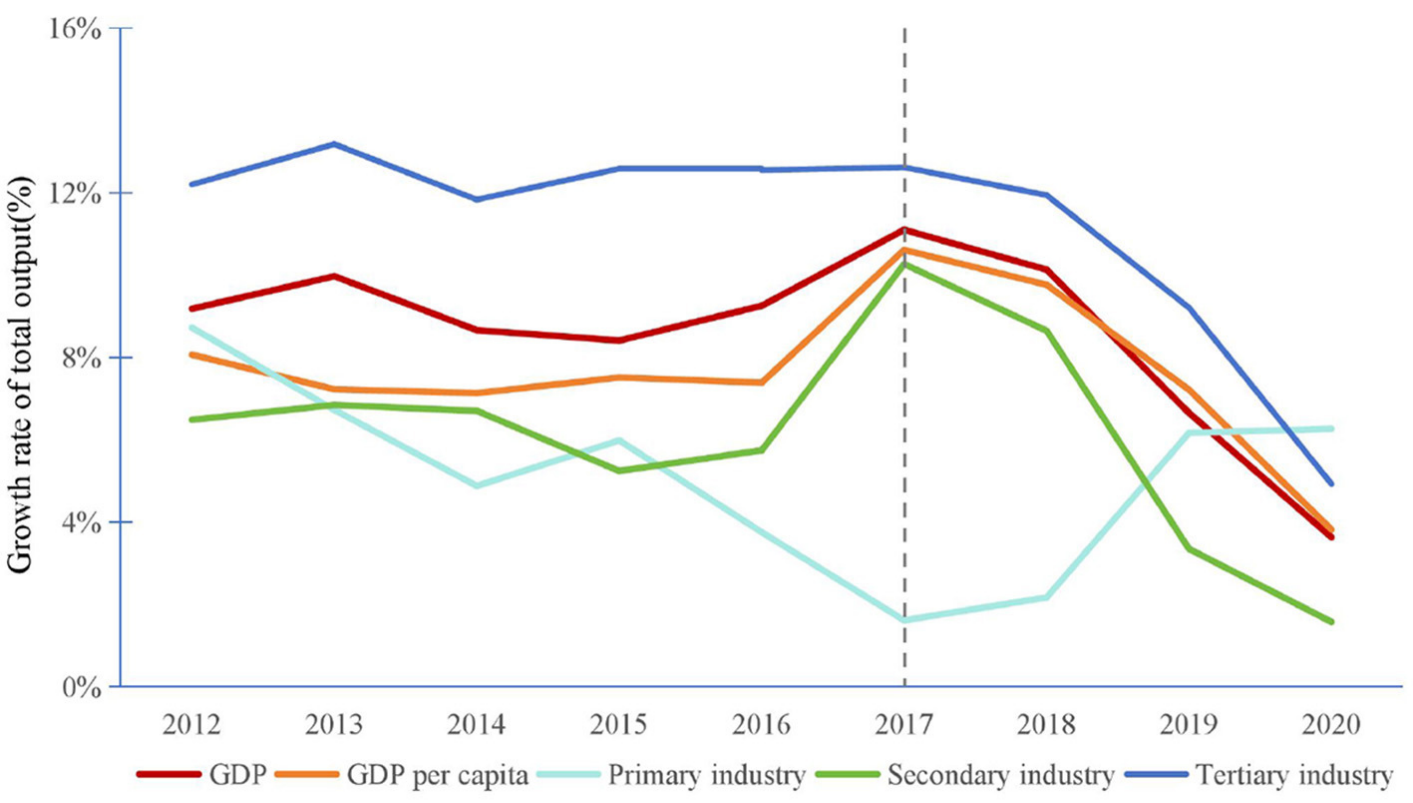
Economic performance of CYRD.

Similar to the study by Fingleton et al. (35) in the UK, this paper distinguishes between two types of economic resilience: recover ability, which refers to the economic recovery ability of cities during a period of stable economic growth, and resistance ability, which refers to the ability to withstand shocks during an economic downturn. According to Figure 3, the GDP of CYRD and the GDP per capita growth rates reached a peak in 2017 and then began to decline. Specifically, the main reason for the slowdown in the economic growth is the declining growth rates of the total output value of the secondary and tertiary industries, which together account for about 90% of the GDP. Therefore, this study sets the period from 2017 to 2020, before the widespread outbreak of the COVID-19 pandemic, as the recessionary period for CYRD, and the period from 2012 to 2017 as the pre-recessionary period.^4^ Additionally, the economic recovery ability is measured by the economic sensitivity index from 2017 to 2019, while economic resistance ability is measured by the economic sensitivity index from 2015 to 2017. The reason for only examining the period from 2015 to 2017, the pre-recessionary period, is to maintain consistency with the length of the recessionary period after 2017. Meanwhile, based on the recovery and resistance indexes, the economic resilience is classified into four types (Table 1).

**Table 1:** Economic resilience classification.

**Groups**	**Types of economic resilience**	**Classification criteria**
Group I	High resistance	Resistance ability >1
Group II	Low resistance	Resistance ability < 1
Group III	Fast recovery	Recovery ability >1
Group IV	Slow recovery	Recovery ability < 1

### 2.3 Econometric model setting

#### 2.3.1 Identification of the effect of internal migrants' health on economic resilience

The effect of internal migrants' health on economic resilience has been supported by theoretical research. To further verify the response of economic resilience to internal migrants' health, this paper constructs the following econometric model:


(2) $$\begin{array}{r} resistance_{i,2017-2019}=\alpha +\beta \;health_{ijt}+\gamma_{1}X_{it}\\ +\gamma_{2}M_{it}+\gamma_{3}Z_{it}+\varepsilon_{it} \end{array}$$

(3) $$\begin{array}{r} recovery_{i,2015-2017}=\alpha +\lambda health_{ijt}+\gamma_{1}X_{it}+\gamma_{2}M_{it}\\ +\gamma_{3}Z_{it}+\varepsilon_{it} \end{array}$$

Where, the dependent variables *resistance* and *recovery* represent the resistance ability index and recovery ability index of city *i*, respectively. The independent variable *health*_*ijt*_ represents the health performance of internal migrants *j* in city *i*. β and λ are the net effects of internal migrants' health on the resistance ability and recovery ability of the urban economic, respectively. *X*, *M*, *Z* are used to control social factors, economic factors, and institutional factors that affect economic resilience.

According to data from China's NBS, the proportion of employment in the three industries for the internal migrants changed from 2.54%, 29.06%, 68.4% in 2015 to 2.36%, 9.28%, 88.36% in 2017, and further to 2.24%, 26.13%, 71.63% in 2018. The employment of the internal migrants is concentrated in the secondary and tertiary industries. Therefore, in addition to examining the overall economic resilience, this paper further distinguishes industry heterogeneity and explores the effect of migrants' health on economic resilience of the secondary and tertiary industries. Furthermore, to overcome potential reverse causality between economic resilience and migrants' health and obtain more robust estimation results, this paper sets the value of t in the econometric model at the initial period. In other words, the t value for Model (3) is set to 2017, and for Model (4) it is set to 2015. This is because the health status during the initial period is considered a predetermined variable, and will not be influenced by subsequent stages of economic resilience.

#### 2.3.2 Identification of the mediating effect of internal migrants' labor supply

How internal migrants' health affects economic resilience is the focus of this paper. Existing literature indicates that labor supply is one of the important factors influencing regional economic vitality and resilience. For CYRD, internal migrants constitute the main body of local labor resource supply, so the economic resilience will be affected by the labor supply status of internal migrants. Specifically, internal migrants' health will indirectly affect the economic resilience by influencing the labor supply of internal migrants.

To test the above-mentioned mediation mechanism, this paper constructs a mediating effect model based on the labor supply of internal migrants. According to the identification strategy, we first construct an econometric Model (5) to test whether the prerequisites for the existence of the mediating effect are met, which is whether the explanatory variable, internal migrants' health, significantly affects the mediator variable, the labor supply of migrants. If the estimation results of Model (5) are prominent, we further construct econometric Models (6) and (8) to test the existence of the mediating effect, that is, to test whether the mediator variable, the labor supply of internal migrants, significantly affects economic resilience. If the mediating effect exists, we finally include both the independent variable and the mediator variable in the baseline regression equation and construct econometric Models (9) and (10) to test the mediation effect of migrants' health on the economic resilience through its effect on the labor supply of internal migrants.


(4) $$\begin{array}{l} labor\text{\_}\sup ply_{ijt}=\alpha +\beta_{\;}health_{ijt}+\gamma_{\;}Z_{it}+\varepsilon_{it} \end{array}$$

(5) $$\begin{array}{r} resistance_{i,2017-2019}=\alpha +\beta_{\;}labor\text{\_}\sup ply_{ijt}+\gamma_{1}X_{it}\\ +\gamma_{2}M_{it}+\gamma_{3}Z_{it}+\varepsilon_{it} \end{array}$$

(6) $$\begin{array}{r} recovery_{i,2015-2017}=\alpha +\lambda_{\;}labor\text{\_}\sup ply_{ijt}+\gamma_{1}X_{it}\\ +\gamma_{2}M_{it}+\gamma_{3}Z_{it}+\varepsilon_{it} \end{array}$$

(7) $$\begin{array}{r} resistance_{i,2017-2019}=\alpha +\beta_{1}health_{ijt}+\beta_{2}labor\text{\_}\sup ply_{ijt}\\ +\gamma_{1}X_{it}+\gamma_{2}M_{it}+\gamma_{3}Z_{it}+\varepsilon_{it} \end{array}$$

(8) $$\begin{array}{r} recovery_{i,2015-2017}=\alpha +\beta_{1}health_{ijt}+\beta_{2}labor\text{\_}\sup ply_{ijt}\\ +\gamma_{1}X_{it}+\gamma_{2}M_{it}+\gamma_{3}Z_{it}+\varepsilon_{it} \end{array}$$

Where, the mediating variables *labor*_sup*ply*_*ijt*_ represent the labor supply performance of internal migrants *j* in city *i*, measured by the individual labor participation rate (*LPR*) and labor time supply of the internal migrant.

#### 2.3.3 Identification of the moderating effect of basic public health services

Existing studies indicate that the health status of the internal migrants is closely related to the accessibility and quality of urban basic public health services. This suggests that adjustments to urban basic public health service policies will indirectly impact the correlation between internal migrants' health and urban economic resilience. To verify this inference and determine the optimal strategy for optimizing urban basic public health services, this paper constructs a moderation effect model. In this model, influencing economic resistance and recovery abilities, an interaction term between basic public health services and internal migrants' health is introduced. The extended econometric model is set as follows:


(9) $$\begin{array}{r} resistance_{i,2017-2019}=\alpha +\beta_{\;}health_{ijt}\\ +\phi_{\;}health_{ijt}\times public\text{\_}service_{ijt}+\gamma_{1}X_{it}+\gamma_{2}M_{it}\\ +\gamma_{3}Z_{it}+\varepsilon_{it} \end{array}$$

(10) $$\begin{array}{r} recovery_{i,2015-2017}=\alpha +\lambda_{\;}health_{ijt}\\ +\varphi_{\;}health_{ijt}\times public\text{\_}service_{ijt}+\gamma_{1}X_{it}+\gamma_{2}M_{it}\\ +\gamma_{3}Z_{it}+\varepsilon_{it} \end{array}$$

Therefore, after introducing the interaction term between urban basic public health service variables and migrants' health variables, the moderating effect coefficients are as follow:


(11) $$\begin{array}{l} \frac{\partial \ln\left(resis\tan ce_{i,2017-2019}\right)}{\partial health_{ijt}}=\beta +\phi \times public\text{\_}service_{ijt} \end{array}$$

(12) $$\begin{array}{l} \frac{\partial \ln\left(re\mathrm{cov}ery_{i,2015-2017}\right)}{\partial health_{ijt}}=\lambda +\varphi \times public\text{\_}service_{ijt} \end{array}$$

In the equations (11) and (12), *public*_*service*_*ijt*_ is the moderating variable. The estimated parameters ϕ and φ characterize the moderating effects of basic public health services on internal migrants' health and the economic resilience. In the above equation, if the estimated parameter ϕ>0, it indicates that enhancing urban basic public services contributes to strengthening the positive effect of migrants' health on economic resilience. If the estimated parameter φ>0, it indicates that enhancing urban basic public services contributes to strengthening the positive effect of migrants' health on urban economic recovery.

### 2.4 Variable section and data sources

**(1)** Dependent variable: The dependent variable is economic resilience, characterized specifically through economic resistance and recovery abilities, as detailed in Section 2.2. The raw data for urban economic resistance and recovery abilities primarily come from various editions of the *China Urban Statistical Yearbook*.

**(2)** Independent variable: The key independent variable is urban internal migrants' health, mainly measured through self-assessment of their health conditions. According to the questionnaire in CMDS, the response options regarding health conditions include “healthy,” “basically healthy,” “unhealthy but able to manage life,” and “unable to manage life”. To facilitate quantitative analysis of migrant health variables, this study uses the Likert scale method to assign values of 4, 3, 2, and 1, respectively, based on the degree of health. The data on migrants' health conditions are sourced from the CMDS, a nationwide annual survey conducted by the National Health Commission of China, concentrating on areas with a significant influx of internal migrants. The survey includes 1,459 county-level units, 3,776 townships, and 8,993 neighborhood committees across the country. CMDS samples nearly 200,000 households each year, covering information on internal migrants, household members, migration patterns, employment, income and housing, health, marriage and fertility, etc.

**(3)** Control variables: In the control variables, social factors include population density (*density*) and its quadratic term, urbanization rate (*urbanization*); economic factors include per capita GDP (*rjgdp*) and its quadratic term, trade dependence rate (*trade*); institutional factors include government expenditure (*expenditure*) and its quadratic term, innovation ability (*innovation*). Population density is measured by the permanent population size per unit area, and urbanization rate is measured by the proportion of urban population. Trade dependence is measured by the proportion of international trade to GDP. Government expenditure level mainly refers to the scale of government fiscal public expenditure, and innovation ability is measured by the number of authorized patents. The raw data for all control variables are also sourced from various editions of *China Urban Statistical Yearbook*, with occasional supplementation from individual city's *Yearbooks* to address missing data.

**(4)** Mediating variable: The mediating variable in this study is the labor supply of internal migrants, primarily measured through two dimensions: individual labor participation rate and labor time supply. Firstly, the individual labor participation rate is measured using a dummy variable, indicating whether internal migrants have participated in work in the past week for at least 1 h with income (*LFP* = *1*) or not (*LFP* = *0*). However, according to Heckman (37), the change in individual labor supply includes both changes in work participation (breadth) and changes in working hours (depth). Therefore, this study also considers the labor time supply variable, measured by the weekly working hours of internal migrants.

**(5)** Moderating variable: The moderating variable in this paper is basic public health services, comprising two dimensions: accessibility and quality. The public health services' accessibility is reflected through indicators such as *health records* (*health_records), health education (health_education)*, etc. The public health services' quality is measured by indicators such as *physical examinations* for major diseases *(Physical_examination), urban medical insurance (medical_insurance)*, etc. For ease of quantitative analysis, all the above variables are set as 0–1 binary dummy variables. Regarding the indicators for basic public health services' accessibility, options such as “heard about the national basic public health service project,” “established resident health records,” and “received health education for major diseases” in the questionnaire are set to 1, while other options are set to 0.^5^ Regarding public health services' quality, options such as “received follow-up assessment and health examination for major diseases” and “participated in urban resident or employee medical insurance” in the questionnaire are set to 1, while other options are set to 0. The original data for the moderating variable of basic public health services are sourced from CMDS.

## 3 Results

### 3.1 Baseline regression

#### 3.1.1 The net effect of internal migrants' health on economic resilience

Based on the identification strategy outlined earlier, this paper decomposes economic resilience into economic resistance and recovery ability, and empirically estimates the overall average effects of internal migrants' health on the economic resilience of CYRD. From the estimation Equations (1, 4) in Table 2, the net effects of internal migrants' health on economic resistance ability and recovery ability are both significantly positive, with marginal coefficients of 0.0186 and 0.0039, respectively. This indicates that migrants' health has a positive effect on economic resilience. Furthermore, for each one unit increase in internal migrants' health, the average of economic resistance ability and recovery ability increases by 0.0186 and 0.0039, respectively. Therefore, enhancing the health level of internal migrants in CYRD has significant effects on improving both economic resistance and recovery abilities. Comparatively speaking, the promotion effect of internal migrants' health on the economic resistance is particularly prominent.

**Table 2:** Baseline regression estimation results.

	**Dependent variable: resistance**			**Dependent variable: recovery**		
	**(1)**	**(2)**	**(3)**	**(4)**	**(5)**	**(6)**
Health	0.0186^***^	0.0287^***^	0.0156^***^	0.0039^*^	0.0053^*^	0.0019
	(0.0009)	(0.0014)	(0.0007)	(0.0021)	(0.0028)	(0.0023)
Density	2.7961^***^	4.8351^***^	1.4841^***^	4.6642^***^	7.6793^***^	2.4146^***^
	(0.0907)	(0.1409)	(0.0677)	(0.3931)	(0.4581)	(0.3882)
Density^*^ density	−9.9479^***^	−9.9813^***^	−2.8623^***^	−23.3391^***^	−35.7448^***^	−8.8158^***^
	(0.4598)	(0.7143)	(0.3429)	(2.0378)	(2.3746)	(2.0124)
Urbanization	1.0037^***^	0.4297^***^	0.1519^***^	−0.0134^***^	−0.0187^***^	−0.0060^***^
	(0.0140)	(0.0217)	(0.0104)	(0.0007)	(0.0008)	(0.0007)
Lnrjgdp	−1.6432^***^	−2.3943^***^	−1.0716^***^	0.3231^***^	0.0028	0.8226^***^
	(0.0137)	(0.0212)	(0.0102)	(0.0586)	(0.0683)	(0.0579)
Lnrjgdp^*^ lnrjgdp	0.3767^***^	0.5715^***^	0.2597^***^	−0.1350^***^	−0.0597^***^	−0.2514^***^
	(0.0032)	(0.0050)	(0.0024)	(0.0154)	(0.0179)	(0.0152)
Trade	−0.0402^***^	−0.1789^***^	−0.0681^***^	−0.0818^***^	−0.1072^***^	−0.0041
	(0.0020)	(0.0030)	(0.0015)	(0.0048)	(0.0055)	(0.0047)
Lnfinance	−0.8760^***^	−1.8247^***^	−0.0261	−0.4994^***^	−0.8545^***^	−0.6401^***^
	(0.0195)	(0.0303)	(0.0145)	(0.0898)	(0.1047)	(0.0887)
Lnfinance^*^lnfinance	0.0539^***^	0.1216^***^	0.0010	0.0484^***^	0.0794^***^	0.0496^***^
	(0.0015)	(0.0024)	(0.0011)	(0.0074)	(0.0087)	(0.0073)
Lninnovation	0.0941^***^	0.1954^***^	0.0101^***^	0.1627^***^	0.1783^***^	0.1505^***^
	(0.0012)	(0.0019)	(0.0009)	(0.0052)	(0.0061)	(0.0052)
_Cons	4.6638^***^	8.3276^***^	1.9174^***^	1.4373^***^	2.8473^***^	−2.4392^***^
	(0.0611)	(0.0950)	(0.0456)	(0.2815)	(0.3281)	(0.2780)
F stats.	5704.1140^***^	3920.3034^***^	4590.4027^***^	384.5057^***^	384.0409^***^	359.8270^***^
R^2^	0.6712	0.6838	0.6216	0.7578	0.7576	0.7454
Obs.	27960	27960	27960	1240	1240	1240

^***^P < 0.01, ^*^P < 0.1, robust standard errors in parentheses. Equations (1, 4) represent the full sample estimation results; Equations (2, 5) represent the estimated results of the secondary industry sample; Equations (3, 6) represent the estimated results of the tertiary industry sample (the same below).

As one of the most dynamic economic regions in China, CYRD accommodates a large number of internal migrants, totaling 73.573 million, accounting for 19.6% of the national internal migrants. The significant presence of internal migrants enriches the labor resources and human capital, driving concentrated economic development and ensuring the sustained and rapid advancement of urbanization. As a crucial component of the labor supply in CYRD, whether internal migrants can provide a continuous labor output becomes an important factor influencing the sustained and stable economic development and resilience of urban agglomerations, with labor supply being constrained by individual health levels. Therefore, enhancing the health level of internal migrants can inject new impetus into economic resilience.

The overall average effect estimation confirms that enhancing internal migrants' health significantly promotes the economic resilience of CYRD. However, since the employment sectors of internal migrants are mainly concentrated in the secondary and tertiary industries, further exploration of the heterogeneous effect of internal migrants' health on the economic resilience of different industries is of practical significance. To address this, this paper replaces the overall economic resilience variable with secondary and tertiary industry economic resilience variables, and re-conducts regression estimations to examine the industry heterogeneity of the effect of migrants' health on economic resistance and recovery abilities. The results of the other estimation equations in Table 2 show that, on the one hand, internal migrants' health positively promotes the economic resistance of both the secondary and tertiary industries, with marginal effects of 0.0287 and 0.0156, respectively. On the other hand, higher migrants' health levels also contribute to the improvement of economic recovery ability in the secondary industry, with a marginal effect of 0.0053. However, the effect of migrants' health status on economic recovery ability of the tertiary industry is not obvious. Comparatively, the effects of migrants' health on economic resistance of both the secondary and tertiary industries are significantly higher than that of economic recovery ability. Additionally, whether from economic resistance or recovery, the secondary industry economic resilience of CYRD is more sensitive to internal migrants' health.

Table 2 also presents the net effects of social, economic, and institutional factors on the economic resilience. From the estimation results of social factors, both for the overall sample and the secondary or tertiary industry samples, population density shows an inverted U-shaped trend in its impact on the economic resistance and recovery abilities. This indicates that increasing population density contributes to enhancing the economic resistance and recovery abilities of the urban agglomeration when population density is low. However, further increases in population density lead to congestion effects, weakening the economic resistance and recovery abilities. Thus, an appropriate level of population density is conducive to economic resilience. Additionally, the increase in urbanization rate has a promoting impact on the economic resistance ability, but suppresses economic recovery ability. The estimation results of economic factor control variables show that the effect of regional economic development level on the economic resistance ability exhibits a U-shaped trend, while its impact on economic recovery ability shows an inverted U-shaped trend. Moreover, the growth of foreign trade dependence is unfavorable for the economic resistance ability together with recovery ability. The estimation results of institutional factor control variables show that there is a U-shaped relationship between government expenditure level and economic resistance ability together with economic recovery ability. Furthermore, increasing government expenditure levels further enhances economic resilience. Innovation capability has a promoting impact on both economic resistance and recovery abilities of CYRD. Finally, the significance tests of the model confirm that the econometric model is well-specified.

#### 3.1.2 Heterogeneity analysis of economic resilience

Section 2.2 divides economic resilience into four groups based on the magnitude of economic resistance and recovery abilities: High resistance, Low resistance, Fast recovery, and Slow recovery. This section further investigates the heterogeneous effect of internal migrants' health on the economic resistance and recovery abilities of CYRD according to different groups of economic resilience. The estimation results in Table 3 demonstrate that, whether for the high economic resistance sample or the low economic resistance sample, internal migrants' health significantly enhances the overall economic resistance as well as economic resistance of the secondary and tertiary industries. However, comparatively, the positive effect of internal migrants' health levels is more prominent in high economic resistance city samples. Table 4 reports the heterogeneous analysis results according to the grouping of economic recovery ability. The estimation results show that the coefficient of the internal migrants' health is only significantly positive in high economic recovery city samples and not significant in low economic recovery city samples, indicating that improving internal migrants' health levels only has a positive effect on high economic recovery city samples. In summary, the heterogeneous effect of internal migrants' health on economic resilience provides guidance for the precise policy-making of local governments.

**Table 3:** Heterogeneity analysis of resistance.

	**High resistance (Resistance ability**>**1)**			**Low resistance (Resistance ability**<**1)**		
	**(1)**	**(2)**	**(3)**	**(4)**	**(5)**	**(6)**
Health	0.0110^***^ (0.0007)	0.0037^***^ (0.0007)	0.0016^***^ (0.0007)	0.0001 (0.0004)	0.0070^***^ (0.0006)	0.0011^***^ (0.0004)
Control	Yes	Yes	Yes	Yes	Yes	Yes
_cons	2.1775^***^ (0.0959)	−4.5903^***^ (0.0863)	3.1543^***^ (0.1542)	14.9872^***^ (0.0861)	−23.7494^***^ (0.6312)	0.6999^***^ (0.0431)
F Stats.	5393.8901^***^	23287.9210^***^	10453.2312^***^	12038.4810^***^	3045.6910^***^	3240.3420^***^
R^2^	0.7417	0.9202	0.7034	0.9294	0.7972	0.7705
Obs.	18,800	20,200	11,873	9,160	7,760	9,660

^***^P < 0.01.

**Table 4:** Heterogeneity analysis of recovery.

	**High recovery (Recovery ability**>**1)**			**Low recovery (Recovery ability**<**1)**		
	**(1)**	**(2)**	**(3)**	**(4)**	**(5)**	**(6)**
Health	0.0025^**^ (0.0012)	0.0051^*^ (0.0027)	0.0009 (0.0014)	0.0003 (0.0014)	0.0002 (0.0017)	0.0001 (0.0011)
Control	Yes	Yes	Yes	Yes	Yes	Yes
_cons	−1.8099^***^ (0.2013)	−2.0022^***^ (0.3077)	−0.0813^***^ (0.1323)	0.4725^***^ (0.0251)	0.1211^***^ (0.0414)	0.1285^***^ (0.0434)
F Stats.	153.6602^***^	129.0410^***^	134.9310^***^	2409.8400^***^	11.650^***^	12.650^***^
R^2^	0.7226	0.7260	0.6662	0.9383	0.2546	0.2705
Obs.	601	498	687	639	282	294

^***^P < 0.01, ^**^P < 0.05, ^*^P < 0.1.

### 3.2 Mediating effect test

Before identifying the mediating effects, the paper first tests the prerequisites for the existence of mediating effects based on the econometric model (5), and then examines the existence of them based on econometric models (6) and (8). The test results in Table 5 show that internal migrants' health has a significant effect on their LPR and labor time supply, meaning that the prerequisites for the existence of mediating effects are met. Specifically, each increase in the health level of internal migrants will effectively increase the labor participation rate by 9% in 2017 and 6% in 2015, while increasing the labor time supply by 1.5% in 2017 and 1.8 in 2015.

**Table 5:** Prerequisite testing for mediating effects.

	**2017**		**2015**	
	**LFP**	**LN (Working _hours)**	**LFP**	**LN (Working _hours)**
	**(1)**	**(2)**	**(3)**	**(4)**
Health	0.0891^***^ (0.0045)	0.0147^***^ (0.0050)	0.0553^***^ (0.0172)	0.0180^**^ (0.0072)
Control	Yes	Yes	Yes	Yes
_cons	0.9691^***^ (0.0229)	4.4508^***^ (0.0150)	1.9171^***^ (0.0911)	4.2844^***^ (0.0991)
F Stats.	559.021^***^	540.020^***^	149.090^***^	9.0900^***^
R^2^	0.7006	0.7111	0.6984	0.6230
Obs.	30000	25928	1358	827

tIn regression Equations (1, 2), individual attribute factors such as gender, age, education, marriage, Hukou status, and other personal characteristics that influence the labor participation rate and labor time supply of the floating population are controlled for. ^***^P < 0.01, ^**^P < 0.05.

Looking at the estimated Equations (1, 2) from Table 6, the labor participation rate and labor time supply of the internal migrants have a positive effect on economic resistance ability, indicating that internal migrants' health does indeed indirectly affect the economic resistance ability by influencing their labor participation rate and labor time supply. Looking at the estimated Equations (5, 6) from Table 6, the labor participation rate and labor time supply of the internal migrants also have a positive effect on economic recovery ability, indicating that internal migrants' health also indirectly affects the economic recovery ability of CYRD through their labor participation rate and labor time supply. In summary, the mediating effects based on the labor participation rate and labor time supply of the internal migrants significantly exist.

**Table 6:** Regression results of mediating effects.

	**Dependent variable: resistance**				**Dependent variable: recovery**			
	**(1)**	**(2)**	**(3)**	**(4)**	**(5)**	**(6)**	**(7)**	**(8)**
Health			0.0174^***^ (0.0009)	0.0228^***^ (0.0109)			0.0036^***^ (0.0013)	0.0020^*^ (0.0013)
LFP	0.0134^***^ (0.0012)		0.0105^***^ (0.0012)		0.0033^***^ (0.0009)		0.0020^*^ (0.0013)	
LN (Working_hours)		0.0086^***^ (0.0013)		0.0092^***^ (0.0013)		0.0050^***^ (0.0011)		0.0055^*^ (0.0036)
Control	Yes	Yes	Yes	Yes	Yes	Yes	Yes	Yes
_cons	4.6717^***^ (0.0614)	4.6256^***^ (0.0667)	4.6526^***^ (0.0611)	4.6033^***^ (0.0662)	0.8768^***^ (0.0494)	0.3587^***^ (0.0552)	1.4398^***^ (0.2816)	1.4610^***^ (0.3483)
F Stats.	5624.600^***^	4808.560^***^	5208.210^***^	4488.800^***^	10571.220^***^	9295.060^***^	349.440^***^	183.820^***^
R^2^	0.6680	0.6647	0.6721	0.6706	0.7454	0.7496	0.7579	0.7326
Obs.	27960	24262	27960	24262	36120	31059	1240	750

^***^P < 0.01, ^*^P < 0.1.

Subsequently, the paper further estimates the mediating effects based on econometric models (9) and (10). The estimated Equations (3, 4) in Table 6 show that, in addition to the direct effect on economic resilience of CYRD, the health of the internal migrants also has a mediating effect on economic resilience through influencing the labor participation rate and labor time supply. Specifically, the mediating effect of the labor participation rate on economic resilience is 0.01, and the mediating effect of labor time supply on economic resilience is 0.9%. Furthermore, according to the estimated Equations (7, 8) in Table 6, in addition to the direct effect on the economic recovery capability, the health of the internal migrants also has a mediating effect on the economic recovery capability through influencing the labor participation rate and labor time supply of internal migrants. Among them, the mediating effect of labor participation rate on economic recovery capability is 0.002, and the mediating effect of labor time supply on economic recovery capability is 0.6%. Thus, it is evident that the mediating effects of the labor participation rate and labor time supply on economic resilience are more pronounced.

### 3.3 Moderating effect test

Since the migrants' health status is related to the basic public health services of the city to which they belong, will basic public health services have a moderating effect on the health and economic resilience? For that, this paper further identifies the moderating effect based on econometric models (11) and (12). The moderating variables are public health services' accessibility and quality. The accessibility of public health services is characterized by residents' health records and health education, while public health services' quality is expressed by physical examinations of major diseases and urban medical insurance. The specific method for estimating the moderating effects is as follows: first, in the baseline regression direction, the interaction terms of internal migrants' health and the public health services' accessibility and quality are added separately for regression estimation; then, the interaction terms are simultaneously included in an analytical framework for regression estimation (Table 7).

**Table 7:** Regression estimation results of moderating effects.

	**Dependent variable: resistance**			**Dependent variable: recovery**		
	**(1)**	**(2)**	**(3)**	**(4)**	**(5)**	**(6)**
Health	0.0098^***^ (0.0015)	0.0055^**^ (0.0023)	0.0067^**^ (0.0030)	0.0035^**^ (0.0015)	0.0075^*^ (0.0040)	0.0059^*^ (0.0034)
Health_records	0.0063^**^ (0.0031)		0.0263^**^ (0.0120)	0.0069^***^ (0.0023		0.0110^***^ (0.0007)
Health_education	0.0052^**^ (0.0024)		0.0053^***^ (0.0009)	0.0423^**^ (0.0218)		0.0110^***^ (0.0007)
Physical_examination		0.0054^***^ (0.0010)	0.0159^*^ (0.0101)		0.0192^***^ (0.0032)	0.0110^***^ (0.0007)
Medical_insurance		0.0035^***^ (0.0009)	0.0016^***^ (0.0009)		0.0078^**^ (0.0033)	0.0110^***^ (0.0007)
Health^*^health_records	0.0001 (0.0026)		0.0129^**^ (0.0069)	0.0029^*^ (0.0016)		0.0039^**^ (0.0017)
Health^*^health_education	0.0150^***^ (0.0020)		0.0029^**^ (0.0013)	0.0098^*^ (0.0061)		0.0105^*^ (0.0063)
Health^*^physical_examination		0.0106^**^ (0.0057)	0.0159^***^ (0.0061)		0.0041^**^ (0.0019)	0.0061^**^ (0.0033)
Health^*^medical_insurance		0.0047 (0.0054)	0.0039 (0.0054)		0.0052^**^ (0.0024)	0.0060^*^ (0.0034)
Control	Yes	Yes	Yes	Yes	Yes	Yes
_cons	4.6776^***^ (0.0652)	4.7733^***^ (0.2676)	5.0269^***^ (0.2881)	1.5026^***^ (0.2826)	2.2471^***^ (0.3550)	2.2809^***^ (0.3599)
F Stats.	3681.3601^***^	234.6330^***^	174.8310^***^	276.2303^***^	124.4710^***^	96.7220^***^
R^2^	0.6746	0.7016	0.7088	0.7594	0.7314	0.7324
Obs.	24871	1412	1312	1240	1655	655

^***^P < 0.01, ^**^P < 0.05, ^*^P < 0.1.

The estimated Equations (1–3) have reported the moderating effects of public health services on the economic resilience. The results indicate consistent estimation results for the interaction terms between internal migrants' health and the accessibility and quality variables of basic public health services in three regression equations mentioned above. Therefore, this paper only analyzes the total regression estimation (Equation 3). On the one hand, the moderating effect mechanism of the basic public health services' accessibility has played a significant role, as enhancing public health services' accessibility helps strengthen the positive effect of internal migrants health on economic resilience. Specifically, the moderation effect of establishing residents' health records is 0.0129, and the moderation effect of conducting health education is 0.0029. On the other hand, the moderating effect mechanism of basic public health services' quality also partly plays a positive moderating role. Implementing crucial illness examinations can significantly enhance the positive effect of internal migrants' health on economic resilience, with a moderation effect of 0.0106. However, increasing urban medical insurance cannot significantly enhance the positive moderating effect of internal migrants' health on economic resilience.

Meanwhile, this paper also estimates the moderating effects of public health services on the economic recovery of CYRD. Considering that the interaction terms between internal migrants' health and public health services' accessibility and quality also yield consistent estimation results in regression (Equations 4 6), this section also only elaborates on the estimation results of Equation (6). Overall, both the mechanisms of the basic public health services' accessibility and quality play positive moderating roles. Enhancing the accessibility and quality of public health services is beneficial for strengthening the positive effect of migrants' health on economic resilience. For basic public health services' accessibility, the moderation effect of establishing residents' health records is 0.0029, and the moderation effect of conducting health education is 0.0098; for public health services' quality, the moderation effect of implementing important disease health examinations is 0.0061, and the moderation effect of increasing urban medical insurance is 0.0060.

## 4 Conclusions

This study deconstructed economic resilience into resistance ability and recovery ability, analyzed the net effect and its heterogeneity of internal migrants' health on economic resilience in CYRD, and further investigated the mediating effect from labor participation rate and labor time supply of the internal migrants, as well as the moderating effect of public health services. The following findings were obtained: firstly, internal migrants' health has a significant positive effect on the economic resilience of CYRD. Specifically, for each unit increase in the health status of internal migrants, the average growth of economic resilience increases by 0.0186, and the average growth of economic recovery increases by 0.0039. Secondly, the net effect of internal migrants health on economic resilience exhibits significant structural differences, industry disparities, and regional heterogeneity. Among them, the impact of internal migrants health on the economic resilience is significantly higher than on economic recovery; the marginal effect of internal migrants health on the resilience of the secondary industry economy is significantly higher than that on the tertiary industry; and cities with high economic resilience and recovery demonstrate a more prominent promotion effect of internal migrants health levels. Thirdly, apart from directly affecting the economic resilience and recovery, the health of internal migrants also exerts indirect effects through their impact on labor force participation rates and labor supply hours, thereby influencing economic resilience and recovery. The mediating effects of labor force participation rates on economic resilience and recovery are 0.01 and 0.002 respectively, while the mediating effects of labor supply hours on economic resilience and recovery are 0.009 and 0.006 respectively. Fourthly, basic public health services have a significant positive moderating effect on the relationship between internal migrants' health and economic resilience. Enhancing the basic public health services accessibility and quality contributes to strengthening the positive effects of internal migrants' health on economic resilience.

Improving the resilience of urban or urban agglomeration economies is not only an intrinsic requirement for enhancing urban competitiveness but also a necessary path to achieve high-quality and sustainable urban development. Currently, external shocks represented by public health emergencies frequently occur, severely affecting the health status and labor supply levels of urban labor forces, thereby weakening the risk resistance capabilities of urban and urban agglomeration economies. Therefore, efforts to enhance urban and urban agglomeration economic resilience should not only focus solely on upgrading economic and industrial structures, optimizing infrastructure, and increasing the number of labor forces but also address health issues among urban labor forces, especially internal migrants. Firstly, community and public health service centers are the most direct, effective, and economical ways for internal migrants to obtain health resources. Establishing health education points in communities and public health service centers can help improve the health awareness and disease prevention capabilities of internal migrants. Secondly, internet digital platforms and face-to-face education should be fully leveraged and new media such as the Internet, WeChat public accounts, and mobile apps can be ways to broaden the channels and methods of health education dissemination. Thirdly, improve the health records management, strengthen the dynamic monitoring of health data for internal migrants, and help them improve their health literacy and health levels. Finally, expand the coverage of important disease health examinations and urban medical insurance to benefit all internal migrants, thereby increasing their health levels while reducing health expenditures. In summary, enhancing the basic public health services can increase labor supply by improving the health levels of internal migrants, thereby consolidating the economic resilience of cities.

## Data availability statement

The original contributions presented in the study are included in the article/supplementary material, further inquiries can be directed to the corresponding author.

## Ethics statement

Ethical review and approval was not required for the study on human participants in accordance with the local legislation and institutional requirements. Written informed consent from the (patients/ participants OR patients/participants legal guardian/next of kin) was not required to participate in this study in accordance with the national legislation and the institutional requirements.

## Author contributions

SZ: Conceptualization, Supervision, Visualization, Writing – original draft. YD: Data curation, Formal analysis, Methodology, Writing – review & editing.

## Footnotes

^1^The data is sourced from the National Bureau of Statistics of China (https://www.stats.gov.cn/sj/sjjd/202302/t20230202_1896485.html).

^2^ http://politics.people.com.cn/n/2013/0204/c70731-20426975.html

^3^ https://m.thepaper.cn/baijiahao_8705887

^4^The reason for not considering the period after 2020, during the COVID-19 pandemic, is that the Chinese and global economies faced significant external shocks during the pandemic, making it difficult to accurately test the impact of migrants' health on economic resilience during this period.

^5^The important diseases in the options mainly include occupational diseases, sexually transmitted diseases, AIDS, tuberculosis, chronic diseases, etc.
